# Knowledge, attitudes, and practices of adolescent girls regarding cervical cancer: a cross-sectional study in Enugu State, Nigeria

**DOI:** 10.11604/pamj.2024.47.17.41087

**Published:** 2024-01-15

**Authors:** Kosisochi Chinwendu Amorha, Gerald Obinna Ozota, Maria-Gorreti Ogechukwu Ndunwere, Ugomma Loveth Anyaji, Ogochukwu Francisca Egbo, Obianuju Anastasia Ogugofor

**Affiliations:** 1 Department of Clinical Pharmacy and Pharmacy Management, Faculty of Pharmaceutical Sciences, University of Nigeria Nsukka, PMB 410001, Enugu State, Nigeria,; 2 Global Health Policy Unit, School of Social and Political Science, University of Edinburgh, Edinburgh, United Kingdom

**Keywords:** Adolescents, attitudes, cervical cancer, knowledge, practice

## Abstract

**Introduction:**

cervical cancer is the second leading cause of cancer death, among women, globally. The majority of the countries with the highest cervical cancer burden are in sub-Saharan Africa, including Nigeria. This study assessed the knowledge, attitudes, and practices regarding cervical cancer among secondary school students in an all-girls school.

**Methods:**

this cross-sectional study was conducted in Nsukka, Enugu State, Nigeria (April 2021). A 30-item self-administered structured questionnaire was filled by conveniently sampled students in Senior Secondary School (SS 2 and SS 3). Pearson's Chi-square was used to test the association between variables (Statistical significance: P < 0.05).

**Results:**

the mean age of the 103 respondents was 16.10 (± 1.00) years. The most common source of information about cervical cancer was mass media (n = 20, 19.4%). Less than half of the respondents had good knowledge of cervical cancer (n = 44; 42.7%) and had favorable attitudes towards the disease (n = 50; 48.5%). More than half of them had good cervical cancer practices (n = 87; 84.5%). They were highly open to screening (n = 92; 89.3%) and vaccination (n = 93; 90.3%). More respondents who had first-hand experience of cervical cancer from family members were aware of the disease compared to those who did not (71.4% Vs. 33.3%; χ^2^= 4.113; P = 0.043).

**Conclusion:**

the study highlights the need for adolescent girls to be educated about cervical cancer, to improve their knowledge and attitudes towards the disease, so they can make informed decisions about their practices.

## Introduction

Cervical cancer is the fourth most common cancer in women and the second leading cause of cancer death, after breast cancer, in women, globally [1,2]. According to the World Health Organization (WHO) 19 of the top 20 countries with the highest cervical cancer burden were in sub-Saharan Africa [1]. As of 2018, Nigeria recorded 14,943 new cervical cancers and 10,403 related deaths accounting for one-fifth of cancer deaths in West Africa [2,3]. Nearly all cases of cervical cancer are caused by the Human Papillomavirus (HPV), acquired mainly through sexual activity [4,5].

The highest rates of HPV infections have been demonstrated to be prevalent among women less than 25 years old [6,7]. In the United States, it has been reported that sexually active adolescents between 14 - 19 years had the highest prevalence of high-risk HPV infection [6]. However, deaths from cervical cancer could be avoided through the HPV vaccination of adolescents [8]. This is further elaborated by the argument that interventions at the university level might be quite late for about 80% of women given that they may have been exposed to HPV [7]. This is underlined by the findings that 81.5% of Nigerian undergraduates are sexually active when compared to 15.6% of 16-year-olds who are mostly in secondary school [7].

Preventing death from cervical cancer is possible when interventions, such as vaccination, are administered before initiating sexual activity; therefore, considered more advantageous when offered during early adolescence [9]. Other approaches include screening tests and treatment which are covered in the WHO global strategy. The triple approach strategy by the WHO (90-70-90 targets), to be met by 2030 for countries on the path to cervical cancer elimination stipulates the following: (i) 90% of girls to be fully vaccinated by 15 years old, (ii) 70% of women to be screened on or before age 35 and screening repeated by age 45, and (iii) 90% of women identified with cervical cancer disease receive treatment [10]. This triple approach strategy is partly based on the Cervical Cancer Elimination Modelling Consortium (CCEMC) model [10].

The CCEMC model, a comparative modeling analysis predicts declines in cervical cancer incidences provided some conditions are met [8]. This model's prediction is as follows: a girls-only HPV vaccination with 90% coverage will lead to an 89.4% reduction in cervical cancer incidence [8]. The addition of screening is also predicted to significantly improve declines in cervical cancer [8]. In terms of years, with vaccination alone, cervical cancer incidence will be halved in low- or middle-income countries (LMICs) by 2061 [8]. With once-lifetime screening, cervical cancer incidence will be reduced by 50% by 2055 [8]. Notably, the models predicted that HPV vaccination with or without screening will reduce cervical cancer incidence in women of childbearing age (< 45 years) by more than 85% before 2050 [8].

Good cervical practices are needed to achieve this, including the acceptability of vaccines and screening [11]. These practices are not inherent but could be learned through education [12-14]. It has been demonstrated that there is an intersectionality between health and education in adolescent girls [12]. Research conducted in Sub-Saharan Africa suggests that poor cervical cancer attitudes and negative attitudes to vaccinations are due to a lack of knowledge about cervical cancer and its prevention, and vice versa [7,15]. In Zambia, for instance, having knowledge and awareness about cervical cancer resulted in positive attitudes towards the HPV vaccine [15]. A cross-sectional study among adolescent girls in Nigeria also demonstrated the relationship between awareness and knowledge of cervical cancer and cervical cancer attitudes [7]. This implies that the education of girls ensures good practices including vaccine and screening acceptability and a subsequent reduction in cervical cancer deaths. Hence, the need for joint information campaigns in this population group to explain the concepts of cervical cancer for improved knowledge and acceptability of the HPV vaccine [12]. Therefore, given this direct link between education and cervical cancer practices in adolescents, this study seeks to assess the knowledge, attitudes, and practices regarding cervical cancer among secondary school students in an all-girls school in Nigeria.

## Methods

**Study design:** this was a cross-sectional study conducted in April 2021.

**Study setting:** the study was conducted among students in Senior Secondary School two and three (SS2 and SS3) in an all-girls secondary school. The single-sex secondary school, a Special Science School, is located in Nsukka, the second largest town in Enugu State. Nsukka is one of the 17 local government areas in Enugu State. The Special Science School was founded by the Anglicans. It is considered a Science School as it focuses mainly on science subjects. The school has boarding facilities with the option for day studentship. It comprises classes for Junior Secondary School and Senior Secondary School. There are three years for Junior Secondary School: JSS 1, JSS 2 and JSS 3. There are also three years for Senior Secondary School: SS 1, SS 2 and SS 3.

**Eligibility criteria:** the inclusion criteria comprised students in Senior Secondary School two and three (SS2 and SS3) who were willing to participate. Students in Junior Secondary School (JSS 1 - JSS 3) and Senior Secondary School 1 (SS 1) were excluded from the study. SS1 students were unavailable, as of the time of the study.

**Sample size and selection:** there were 282 students in SS2 and SS3. This consisted of 175 and 107 students in SS2 and SS3, respectively. The minimum sample size required for the study was determined with the aid of the Raosoft® sample size calculator. The sample size was calculated to be 163, with a 95% confidence level, a 5% margin of error, and assuming a 50% response distribution. A 200-capacity Hall was provided for the study. In the Hall, one hundred and thirty-six (136) eligible students were seated. Those who provided consent were conveniently sampled and recruited for the study.

**Data collection:** the study utilized a 30-item self-administered structured questionnaire comprising five sections. The first section sought demographic information. The second section was a 2-item scale that sought awareness about cervical cancer. The third section, a 10-item scale, assessed knowledge of cervical cancer with options of ´true´ (coded as ´1´), ´false´ (coded as ´2´), and ´not sure´ (coded as ´3´). The scoring of statements was transformed to binary responses of ´correct´ or ´incorrect´. Items that had ´true´ or ´false´ as correct options were transformed to ´correct´ (coded as ´1´) while those that were ´incorrect´ were coded as ´0´. All ´not sure´ options were transformed to ´incorrect´ (coded as ´0´). The fourth section was a 9-item scale that assessed attitudes towards cervical cancer on a five-point Likert scale of ´strongly disagree´ (coded as 1), ´disagree´ (coded as 2), ´neutral´ (coded as ´3´), ´agree´ (coded as ´4´), and ´strongly agree´ (coded as ´5´). The scoring of the negatively-worded statements was reversed to ´strongly disagree´ (coded as ´5´), ´disagree´ (coded as ´4´), ´neutral´ (coded as ´3´), ´agree´ (coded as ´2´), and ´strongly agree´ (coded as ´1´), during analysis. Increasing scores indicated more favourable attitudes towards cervical cancer, as they understood the threat posed by the disease and were willing to handle it. The last section was a 4-item scale that focused on practice regarding cervical cancer. The students provided information on the actions they had taken or would take regarding cervical cancer.

The questionnaire items were drafted by two clinical pharmacists with experience in cervical cancer settings. Other clinical pharmacists were given the questionnaire for content validation. The questionnaire was piloted among 10 conveniently sampled secondary school students in SS2 and SS3. This did not only eliminate ambiguities but also determined the approximate time it would take to fill out the questionnaire. Data obtained during the pilot test were excluded from the study data. In the Hall, the eligible students who provided consent for participation filled out the questionnaire without consulting any reference material or the Internet. There was confidentiality as the names of the students were not requested. After the filled questionnaires were retrieved, the researchers educated the students on cervical cancer via PowerPoint presentation. The presentation covered all aspects of the questionnaire. There were slides on the definition of cervical cancer, stages of cervical cancer, signs and symptoms of cervical cancer, causes of and risk factors for cervical cancer, prevention of cervical cancer, treatment of cervical cancer, myth vs. fact. These were aimed at improving the knowledge of cervical cancer and addressing misconceptions about the disease. After the PowerPoint presentation, there was a question-and-answer session where other concerns were addressed.

**Data analysis:** data were analyzed using IBM SPSS Version 25.0. Data were summarized using descriptive statistics (frequency, percentages, mean, standard deviation). Pearson´s Chi-square test was used to test the association between the demographic variables, awareness, knowledge, attitudes, and practice regarding cervical cancer. Statistical significance was set as P < 0.05.

**Ethical approval:** the study protocol was approved by the Health Research and Ethics Committee (HREC) of the University of Nigeria Teaching Hospital (UNTH), Ituku-Ozalla, Enugu State on 15^th^ February 2021 (UNTH/HREC/2021/02/300). Permission was received from the Principal of the School to have interactions with the students. The students were informed that participation was voluntary. The students provided consent for participation (written and oral).

## Results

One hundred and three (103) students participated in the study. As depicted in Table 1, the majority of the respondents were between 15 - 17 years old (n = 90; 87.3%). The mean age of respondents was 16.10 (± 1.00) years. About a quarter of the respondents were in SS3 (n = 24; 23.3%). Less than a tenth of the respondents had first-hand experience of cervical cancer from family members who had the disease (n = 7; 6.8%). The most common source of information about cervical cancer was mass media (n = 20, 19.4%). Less than half of the respondents knew that human papillomavirus (HPV) is most responsible for cervical cancer (n = 44; 42.7%) and cervical cancer occurs only in women (n = 51; 49.5%). Overall, less than half of the respondents had *good knowledge* about cervical cancer (n = 44; 42.7%) (Table 2). Three-quarters of the respondents agreed that cervical cancer can be fatal (n = 78; 75.8%) and were favorably disposed to screening for the disease so it can be treated if detected (n = 77; 74.8%). Overall, less than half of the respondents had *favorable attitudes*toward cervical cancer (n = 50; 48.5%) (Table 3).

**Table 1:** demographic characteristics of study participants, recruited from an all-girls secondary school in Nsukka, Enugu State, South-East, Nigeria, in April 2021 (N = 103)

Variables	n (%)
**Age (in years)**	
13	1 (1.0)
14	4 (3.9)
15	16 (15.5)
16	55 (53.4)
17	19 (18.4)
18	6 (5.8)
19	2 (1.9)
**Class**	
SS2	79 (76.7)
SS3	24 (23.3)
**I have a family member who is a health professional (e.g. medical doctor, pharmacist, nurse)**	
Yes	60 (58.3)
No	43 (41.7)
**I have a family member (immediate or extended) who has/had cervical cancer**	
Yes	7 (6.8)
No	96 (93.2)
**Place of upbringing**	
Urban	90 (87.4)
Rural	13 (12.6)

Mean age (years) ± standard deviation = 16.10 ± 1.00; SS = senior secondary school

**Table 2:** awareness and knowledge of cervical cancer among study participants, recruited from an all-girls secondary school in Nsukka, Enugu State, South-East, Nigeria, in April 2021 (N = 103)

Variables	n (%)
	
**Awareness about cervical cancer**	
I have heard about cervical cancer	37 (35.9)
**Sources of information about cervical cancer**	
1. Parents	15 (14.6)
2. Teachers	9 (8.7)
3. Classmates	7 (6.8)
4. Mass media	20 (19.4)
5. Internet	10 (9.7)
6. Health professionals	14 (13.6)
	**Correct (%)**
**Knowledge of cervical cancer**	
1. † The cervix is a part of the digestive system	56 (54.4)
2. Human papillomavirus is most responsible for cervical cancer	44 (42.7)
3. † Cervical cancer is more common in men than women	51 (49.5)
4. Having many sexual partners is a risk factor for cervical cancer	72 (69.9)
5. Smoking is not a risk factor for cervical cancer	60 (58.3)
6. † It is not possible to screen for cervical cancer	66 (64.1)
7. In early-stage cervical cancer, there are no signs/symptoms	35 (34.0)
8. In advanced cervical cancer, there could be pain during intercourse	63 (61.2)
9. Cervical cancer is treatable if detected early	77 (74.8)
10. † A 50-year-old woman does not need pap smears due to her age	42 (40.8)

† The correct option is reversed; the total knowledge score was the sum of the correct options for the items testing knowledge of cervical cancer, with a maximum total knowledge score of 10; knowledge was categorized as good or poor such that good knowledge of cervical cancer referred to those with total knowledge scores > 6 (the median score); less than half of the respondents had good knowledge about cervical cancer (n = 44; 42.7%).

**Table 3:** attitudes of study participants, recruited from an all-girls secondary school in Nsukka, Enugu State, South-East, Nigeria, in April 2021, towards cervical cancer (N = 103)

Items	n (%)				
	SD	D	N	A	SA
1. † I can never have cervical cancer since I do not have a family history of the disease	21 (20.4)	37 (35.9)	15 (14.6)	12 (11.7)	18 (17.5)
2. I would like to screen for cervical cancer so it can be treated if detected	3 (2.9)	6 (5.8)	17 (16.5)	59 (57.3)	18 (17.5)
3. † Screening for cervical cancer is an expensive test	3 (2.9)	13 (12.6)	57 (55.3)	27 (26.2)	3 (2.9)
4. † It is unnecessary to screen for cervical cancer if there are no signs and symptoms	19 (18.4)	45 (43.7)	17 (16.5)	19 (18.4)	3 (2.9)
5. Cervical cancer could lead to death	5 (4.9)	3 (2.9)	17 (16.5)	53 (51.5)	25 (24.3)
6. † It is impossible to screen for cervical cancer in Nigeria as we lack the facilities	11 (10.7)	27 (26.2)	45 (43.7)	15 (14.6)	5 (4.9)
7. † I cannot encourage others to screen for cervical cancer as it is the responsibility of health professionals	20 (19.4)	48 (46.6)	21 (20.4)	10 (9.7)	4 (3.9)
8. † Cervical cancer and other cancers are spiritual problems	43 (41.7)	37 (35.9)	20 (19.4)	2 (1.9)	1 (1.0)
9. † I will not do cervical cancer screening unless it is free of charge	23 (22.3)	26 (25.2)	42 (40.8)	9 (8.7)	3 (2.9)

Strongly Disagree (coded as ‘1’); Disagree (coded as ‘2’); Neutral (coded as ‘3’); Agree (coded as ‘4’); Strongly agree (coded as ‘5’); † Reversed Items such that: Strongly Disagree (coded as ‘5’); Disagree (coded as ‘4’); Neutral (coded as ‘3’); Agree (coded as ‘2’); Strongly agree (coded as ‘1’); the total attitude score was the sum of the scores for the different items with a maximum total attitude score of 45; attitudes were categorized as favourable or unfavourable such that favourable attitudes toward cervical cancer referred to those with a total attitude score > 32 (the median score); less than half of the respondents had favourable attitudes toward cervical cancer (n = 50; 48.5%)

Few respondents (n = 2; 1.9%) had previously been screened for cervical cancer. However, the majority of the respondents reported that they would like to be screened for cervical cancer (n = 92; 89.3%) and would encourage others to do the same (n = 101; 98.1%). Only a tenth did not want to be vaccinated against cervical cancer (n = 10; 9.7%). Overall, more than half of the respondents had good cervical cancer practices (n = 87; 84.5%) (Table 4). A large proportion of respondents who had first-hand experience of cervical cancer from family members were aware of the disease compared to those who did not have a family member with cervical cancer (71.4% Vs. 33.3%; χ^2^= 4.113; P = 0.043). A large proportion of students in SS2 had favourable attitudes towards cervical cancer compared to those in SS3 (54.4% Vs. 29.2%; χ^2^= 4.704; P = 0.030) (Table 5).

**Table 4:** practice of study participants, recruited from an all-girls secondary school in Nsukka, Enugu State, South-East, Nigeria, in April 2021, regarding cervical cancer (N = 103)

Variables	n (%)
**I have previously been screened for cervical cancer (e.g. pap smear test)**	
Yes	2 (1.9)
No	101 (98.1)
**I would like to be screened for cervical cancer**	
Yes	92 (89.3)
No	11 (10.7)
**I would encourage others to screen for cervical cancer**	
Yes	101 (98.1)
No	2 (1.9)
**I would like to take the vaccine for cervical cancer (HPV vaccine)**	
Yes	93 (90.3)
No	10 (9.7)

Yes (coded as ‘1’); no (coded as '0’); the total practice score was the sum of the scores for the different items with a maximum total practice score of 4; the practice was categorized as good or poor such that good practices regarding cervical cancer referred to those with total practice score ≥ 3 (the median score); more than half of the respondents had good cervical cancer practices (n = 87; 84.5%)

**Table 5:** association between demographic variables, awareness, knowledge, attitudes, and practice regarding cervical cancer of study participants, recruited from an all-girls secondary school in Nsukka, Enugu State, South-East, Nigeria, in April 2021, (N = 103)

Variables	Awareness		χ^2^	P	Knowledge		χ^2^	P	Attitudes		χ^2^	P	Practice		χ^2^	P
	Yes	No			Poor	Good			UA	FA			Poor	Good		
**Age (in years)**			8.453	0.207			6.729	0.347			12.206	0.058			7.250	0.298
13	1 (100.0)	0 (0.0)			0 (0.0)	1 (100.0)			0 (0.0)	1 (100.0)			0 (0.0)	1 (100.0)		
14	2 (50.0)	2 (50.0)			4 (100.0)	0 (0.0)			2 (50.0)	2 (50.0)			0 (0.0)	4 (100.0)		
15	8 (50.0)	8 (50.0)			8 (50.0)	8 (50.0)			3 (18.8)	13 (81.3)			3 (18.8)	13 (81.3)		
16	17 (30.9)	38 (69.1)			30 (54.5)	25 (45.5)			30 (54.5)	25 (45.5)			8 (14.5)	47 (85.5)		
17	5 (26.3)	14 (73.7)			12 (63.2)	7 (36.8)			13 (68.4)	6 (31.6)			2 (10.5)	17 (89.5)		
18	2 (33.3)	4 (66.7)			3 (50.0)	3 (50.0)			3 (50.0)	3 (50.0)			3 (50.0)	3 (50.0)		
19	2 (100.0)	0 (0.0)			2 (100.0)	0 (0.0)			2 (100.0)	0 (0.0)			0 (0.0)	2 (100.0)		
**Class**			0.034	0.854			0.348	0.555			4.704	0.030*			2.137	0.144
SS2	28 (35.4)	51 (64.6)			44 (55.7)	35 (44.3)			36 (45.6)	43 (54.4)			10 (12.7)	69 (87.3)		
SS3	9 (37.5)	15 (62.5)			15 (62.5)	9 (37.5)			17 (70.8)	7 (29.2)			6 (25.0)	18 (75.0)		
**A4**			2.060	0.151			1.852	0.174			1.320	0.251			1.638	0.201
Yes	25 (41.7)	35 (58.3)			31 (51.7)	29 (48.3)			28 (46.7)	32 (53.3)			7 (11.7)	53 (88.3)		
No	12 (27.9)	31 (72.1)			28 (65.1)	15 (34.9)			25 (58.1)	18 41.9)			9 (20.9)	34 (79.1)		
**A5**			4.113	0.043*			0.639	0.424			0.222	0.637			0.009	0.925
Yes	5 (71.4)	2 (28.6)			3 (42.9)	4 (57.1)			3 (42.9)	4 (57.1)			1 (14.3)	6 (85.7)		
No	32 (33.3)	64 (66.7)			56 (58.3)	40 (41.7)			50 (52.1)	46 (47.9)			15 (15.6)	81 (84.4)		
**Place of upbringing**			1.067	0.302			0.868	0.351			0.167	0.682			0.697	0.404
Urban	34 (37.8)	56 (62.2)			50 (55.6)	40 (44.4)			47 (52.2)	43 (47.8)			15 (16.7)	75 (83.3)		
																
Rural	3 (23.1)	10 (76.9)			9 (69.2)	4 (30.8)			6 (46.2)	7 (53.8)			1 (7.7)	12 (92.3)		

* P < 0.05 is statistically significant; SS = senior secondary school; A4 = I have an immediate family member who is a health professional; A5 = I have a family member who has or had cervical cancer; UA = unfavourable Attitude; FA = favourable attitude

## Discussion

The findings of this study highlight the need for adolescent girls to be educated about cervical cancer, to improve their knowledge and attitudes towards the disease, so that they can make informed decisions about their practices. The majority of the participants were between 15 - 17 years old. About a quarter of the respondents were in SS3. This is similar to two cross-sectional studies conducted among adolescent girls in Brazil and Nigeria. The mean ages of adolescents sampled in both studies were 12 and 16 years, respectively [6,7]. The age range for the studies appears to be based on the definition of an adolescent. The WHO defines adolescence as the phase of life between childhood and adulthood from ages 10 - 19 [16].

Cervical cancer awareness was poor. Mass media was the major source of information. Less than half of the respondents knew that human papillomavirus (HPV) was most responsible for cervical cancer and that cervical cancer occurs only in women. Overall, less than half of the respondents had good knowledge about cervical cancer. This was similar to a cross-sectional study undertaken among adolescent girls in South-East Nigeria with less than 50% of the participants reported to have *good knowledge* about cervical cancer [7]. In the same study, mass media was reported as the main source of information and knowledge about cervical cancer [7]. In contrast, another cross-sectional study assessing the knowledge of adolescents on cervical cancer demonstrated that more than half of the respondents had good knowledge about cervical cancer [12]. Additionally, the main sources of information were schools, with health professionals coming second [12]. The differences in these studies suggest that there is an influence the source of knowledge on cervical cancer has on the correctness of the information on cervical cancer.

Three-quarters of the respondents agreed that cervical cancer can be fatal and were favorably disposed to screening for the disease so it can be treated if detected. Overall, less than half of the respondents had *favorable attitudes* toward cervical cancer. This is similar to a longitudinal cohort study where it was demonstrated that 27% of the sampled adolescents had an unfavourable attitude toward cervical cancer, specifically, the willingness to accept HPV vaccination [17]. In contrast, another cross-sectional study carried out in Brazil revealed that the sampled adolescents showed good acceptance of the HPV vaccines, more than half of the respondents had positive attitudes toward cervical cancer and were open to screening at least once every three years [12]. In contrast, findings from another study revealed that the extent of positive behaviour and attitudes of adolescents toward cervical cancer were solely dependent on the level of education of the adolescents´ parents. None of these studies provided information on the knowledge of the fatality of cervical cancer among adolescents. On the other hand, another cross-sectional study in Mozambique demonstrated that adolescents´ willingness to be vaccinated to prevent cervical cancer was due to a good understanding and perception of the purpose of vaccination to prevent cervical cancer [18].

A large proportion of respondents who had first-hand experience of cervical cancer from family members were aware of the disease compared to those who did not have family members with cervical cancer. A similar study in Italy demonstrated that having a personal, familiar, or friendly history of cervical cancer made young girls more knowledgeable about cervical cancer [9]. A large proportion of students in SS2 had favourable attitudes toward cervical cancer compared to those in SS3. The results from this study seem to indicate that older adolescents with higher education levels had a less favourable attitudes than younger adolescents. This was similar to a cross-sectional study done in Zimbabwe that showed that a higher level of education did not determine the differences in cervical cancer knowledge [19]. In this case, high school students showed more knowledge about cervical cancer when compared to adolescent students in the university [19]. In contrast, however, other studies showed that adolescents who were older and/or had higher education levels had a more favourable attitudes toward cervical cancer [9,18].

This study had some limitations. It was cross-sectional by design and conducted among females in a senior secondary school in South-Eastern Nigeria. This limits the generalisability of the findings. Other limitations were the poor participation of eligible students, especially the SS3 students who were preparing for their Senior School Certificate Examinations. Although the study was conducted in an all-girls secondary school, all genders must be aware and knowledgeable about cervical cancer. Since males can transmit the virus to females who present with cervical cancer, it is pertinent that males understand how to prevent cervical cancer and reduce the burden of the disease.

## Conclusion

The study highlights the need for adolescent girls to be educated about cervical cancer, to improve their knowledge and attitudes towards the disease, so they can make informed decisions about their practices.

### What is known about this topic

- *Cervical cancer is the fourth most common cancer in women and the second leading cause of cancer death, after breast cancer, in women, globally;*
- *As of 2018, Nigeria recorded 14,943 new cervical cancers and 10,403 related deaths accounting for one-fifth of cancer deaths in West Africa;*
- *The highest rates of HPV infections have been demonstrated to be prevalent among women less than 25 years old; in the United States, it has been reported that sexually active adolescents between 14 - 19 years had the highest prevalence of high-risk HPV infection; interventions at the university level might be quite late*.

### What this study adds

- *Adolescent girls could have deficient knowledge of cervical cancer, with the need for their attitudes towards the disease to be improved; due to the high prevalence of the disease among women less than 25 years old, targeted educational programmes could be designed for adolescents to prevent the burden of cervical cancer due to poor decisions;*
- *The high reception of the respondents to screening for cervical cancer and vaccination is a pointer that if these services are provided, they would be willing to access them; public health enthusiasts, including the government and non-governmental organizations, could organize regular programmes on training, re-training, screenings, and vaccinations amongst this important age category*.
